# Muramyl Tripeptide Plus Chemotherapy Reduces Metastasis in Non-Metastatic Osteosarcoma: A Single-Center Experience

**DOI:** 10.31557/APJCP.2020.21.3.715

**Published:** 2020-03

**Authors:** Nurdan Taçyıldız, Emel Ünal, Handan Dinçaslan, H Mine Çakmak, Kenan Köse, Gülşah Tanyıldız, Ömer Kartal

**Affiliations:** 1 *Division of Pediatric Hematology and Oncology, Department of Pediatrics, *; 2 *Department of Biostatistics, Medical University of Ankara, *; 3 *Division of Pediatric Hematology and Oncology, Department of Pediatrics, Gülhane Training and Research Hospital, Ankara, Turkey. *

**Keywords:** Osteosarcoma, treatment, mifamurtide, metastasis, retrospective study

## Abstract

**Background:**

The immunomodulator mifamurtide plus a chemotherapy regimen has been shown to significantly improve the outcome in non-metastatic osteosarcoma patients. We report the results of the addition of mifamurtide to chemotherapy in newly diagnosed patients with osteosarcoma.

**Methods:**

A total of 36 children with osteosarcoma without detectable metastasis were treated between November 2010 and April 2018 at the Ankara University Department of Pediatric Oncology. Mifamurtide was added to the chemotherapy regimen in 17 patients while the remaining 19 did not receive mifamurtide. The probabilities of metastasis and overall survival were compared between the groups.

**Results:**

The 43-month survival rate was 87.5% and 89.9% in the patients who received and did not receive mifamurtide, respectively (p=0.65). Common side effects of mifamurtide were chills and fever. The addition of mifamurtide in the high-risk group with ≤95% necrosis tended to decrease the probability of distant metastasis (36.4% vs. 58.3%) (p=0.39). The time to metastasis in the group with positive surgical margins (4 months in one patient in the non-mifamurtide group, 7 and 20 months in the mifamurtide group) was also longer in the mifamurtide group. During the 43-month follow up period, median time to metastasis was longer in the mifamurtide group (20 vs. 5 months). In addition, mifamurtide plus chemotherapy decreased the risk of metastasis in the cases with primary site relapse.

**Conclusions:**

The addition of mifamurtide to chemotherapy might improve event-free survival by decreasing the probability of distant metastasis in bad histologic responders, and also by increasing the time to distant metastasis in the surgical margin positive group. Additional clinical studies are necessary to determine the long-term effects of mifamurtide on metastatic disease.

## Introduction

Muramyltripeptidephosphatidyl ethanolamine (MTP-PE) is a synthetic derivative of muramyl dipeptide and is produced from the immunostimulatory components of Gram-positive and Gram-negative bacterial cell walls (Nardin et al., 2006). The intravenous liposomal formulation of MTP-PE (mifamurtide) targets monocytes and macrophages, followed by the phagocytosis of mifamurtide. MTP-PE is a specific ligand of the nucleotide-binding oligomerization domain 2 (NOD2) receptor, leading to the stimulation of nuclear factor-KB. L-MTP-PE, which interacts with (IFN)-gamma, upregulates tumoricidal activity (Ando et al., 2011). 

L-MTP-PE was initially developed as an immunostimulant with significant anti-tumor effects in preclinical models (Fidler et al., 1981; Nardin et al., 2006).

In initial analyses using the nonmetastatic osteosarcoma cohort of INT 0133, the addition of mifamurtide to traditional chemotherapy did not lead to a survival advantage (Meyers et al., 2005). In addition, with longer follow-up of the same cohort in the COG (Children’s Oncology Group) study, a significant interaction between L-MTP-PE and ifosfamide could not be shown. However, the event-free survival (EFS) was longer and there was a statistically significant improvement in overall survival (Meyers et al., 2008; Whelan et al., 2015).

Late diagnosis increases the risk of metastases which is still an important problem in all developing countries including ours. The question is whether the addition of mifamurtide would improve the EFS of patients at our center. We report our results in patients who had no metastasis initially and received mifamurtide and chemotherapy. 

## Materials and Methods

The patients had been histologically diagnosed as intramedullary OS by biopsy. Among the 36 patients with no initially detectable metastases, 17 received L-MTP-PE plus chemotherapy while 19 only received the chemotherapy regimen. The age at enrollment ranged from 7.6 to 16.5 years with a median of 12.1 years. There were 16 female and 20 male patients. All patients received neo-adjuvant chemotherapy including the regimens in Table 1 and had no initially detectable metastasis. All had sufficient renal function (serum creatinine ≤1.5 x normal or creatinine clerance ≥70 ml/min/1.73 m²), hepatic function (total bilirubin ≤1.5 x normal and ALT or AST ≤1.5 x normal), and cardiac function (shortening fraction ≥ 28% or ejection fraction ≥ 50%), to receive anthracyclines.

*Treatment*

All patients received high-dose methotrexate (HDMTX)-based chemotherapy and most patients (69.4%) received the Euramos chemotherapy protocol. This protocol contains two regimens (MAP and MAP + IE) (Whelan et al., 2015) (Table 1).

MTP treatment began after the operation (amputation or resection) and the wound healing period. The dose was 2 mg/m2/day twice a week by intravenous infusion in the first 12 weeks, followed by administration once a week at the same dose for 24 weeks (Anderson et al., 2010).

The clinical status was monitored every 3 months by CT (computerized tomography) and primary region MRI (magnetic resonance imaging). PET-CT (positron emission tomography) was also performed in addition to thoracic CT and primary region MRI preoperatively and at the end of treatment.

*Statistical Methods*

A total of 36 children suffering from osteosarcoma without detectable metastasis were treated with a chemotherapy regimen containing high-dose methotrexate between November 2010 and April 2018 at the Ankara University School of Medicine’s Department of Pediatric Oncology.

The survival rates of the treated with and without MTP-PE were compared.

The overall survival (OS) from the first visit until death or last contact was analyzed. The events were defined as metastases and primary tumor recurrences. The longest follow-up period was 48 months in the mifamurtide group. We therefore compared patients receiving and not receiving mifamurtide using the data from the first 48 months. All statistical analyses were performed with the Statistical Packages for Social Sciences (SPSS), version 11.5 (Chicago Inc.) (SPSS Base, 2003 ).

Overall survival was estimated with the Kaplan-Meier survival analysis and the significance of the comparisons of risk for the adverse event determined with the log-rank test. The survival and metastasis rates were calculated for the period from the date of first presentation to April 2018. The log-rank test was also used for the comparison of the Euramos chemotherapy arms (MAP and MAP+IE). 

The Chi-square test and Fisher’s exact test were used to evaluate the rates. A p value ≤0.05 was accepted as significant.

## Results

The median follow-up time was 36 months with a range of 7-133 months. The femur was the primary tumor site in 27 (75%) patients (Table 2).

*Outcome*

L-MTP-PE was first added to chemotherapy regimens in January 2013 at our pediatric oncology clinic. A total of 4 patients (11.1%) died within the 43 months. 

*Overall Survival*

The overall survival rate in our cohort was 88.9% at 43 months from study entry.

*Side Effects*

The most common side effects were chills and fever (14/17). Other side effects included headache in 6 patients (35%), back pain in 4 patients (23.5%), arthralgia in 4 patients (23.5%), and tinnitus in 1 patient (6%). There were 6 (35%) patients with both fever and headache. Arrhythmia (tachycardic) developed in one patient. All of the side effects disappeared within several hours after the infusion. Antihistamines and antipyretics were useful as premedication and for the treatment of chills, fever and pain.

*Outcome by Treatment Study Arm*

**Figure 1:**
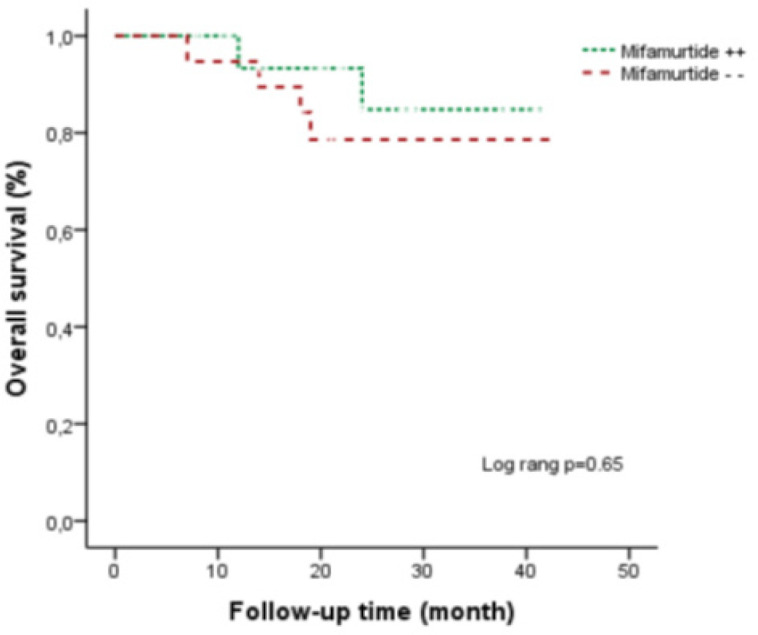
OS at 43 Months was Similar in the Mifamurtide Positive and Negative Groups (p=0.65). However, the mifamurtide positive groups had a trend towards better OS at the 40^th^ month

The 43-month survival rate was 87.5% in the mifamurtide group and 89.9% in the non-mifamurtide group (p=0.65) (Figure 1). There were 4 (11.1%) patients with primary site recurrence; 2 (11.7%) of them were in the mifamurtide group and 2 (10.5%) were in the non-mifamurtide group. 

Overall survival was 90.0% for all patients treated with Euromos MAP and 85.7% for those treated with the Euramos MAP+IE, with no significant difference between the two chemotherapy regimens (p=0.593). Median time to metastasis was 48 (41-55) months in the Euromos MAP arm and 30 (25.8-34.2) months in the Euramos MAP+IE arm (p=0.62). The survival and metastasis parameters were therefore similar in the MAP and MAP+IE arms of the Euramos chemotherapy protocol in our study.

*Necrosis and mifamurtide*

All of the patients underwent definitive resection after induction. Necrosis evaluation was performed by the pathologist. The necrosis value was >95% in 9 patients (27.8 %) and ≤95% in 24 patients (66.7 %) while 3 patients’ necrosis value (5.5%) was not reported. In the mifamurtide group with a necrosis ratio of >95%, 1 (20%) patient developed metastasis. In the non-mifamurtide group with a necrosis ratio of >95%, similarly 1 (25%) patient developed metastasis. In the group of patients with ≤95% necrosis, metastasis was detected in 4 patients (36.4%) in the mifamurtide group and 7 (53.9%) in the non-mifamurtide group (p=0.39) (Table 3).

*Surgical Margins*

Metastasis developed during follow-up in all 3 patients positive for surgical margins but in only 10 (33%) of the patients negative for surgical margins. The difference was statistically significant (p=0.05). Positive surgical margins were present in 2 patients in the mifamurtide group (these patients developed metastasis at the 7 and 20^th^ months, respectively) and in 1 patient in the non-mifamurtide group (the patient developed metastasis at the 4^th^ month). Mifamurtide tended to delay the development of metastasis.

*Primary tumor recurrence*

The median time to pulmonary metastasis was shorter in the primary tumor recurrence group than in the non-recurrent osteosarcoma group (25.6±9.2 vs. 89±10.9 months, respectively), and the difference was statistically significant (p=0.037).

During the 45-month follow-up, there were 4 primary tumor recurrences and 2 of these were in the mifamurtide group. One of the patients with primary tumor recurrence in the mifamurtide group had pulmonary metastatic disease development at the 18^th^ month. Metastasis was detected in two patients in the non-mifamurtide group; one of these had metastasis development at the 14^th^ month and the other at the 45^th^ month. 

*Mifamurtide and Time to metastasis*

During the 43-month follow-up period, median time to metastasis was 20 months (20.9 ±10.45) in the mifamurtide group and 5 months (15.00±15.83) in the non-mifamurtide group (p=0.22). 

Metastases in the first 6 months were seen in 1 (5.8%) patient in the mifamurtide group and 4 (21%) patients in the non-mifamurtide group, with no significant difference (p=0.22).

## Discussion

Approximately 80-90% of newly-diagnosed osteosarcoma patients without detectable metastatic disease are assumed to have micrometastatic disease, which is subclinical or undetectable using current diagnostic modalities (Luetke et al., 2014). Mifamurtide, as an immunomodulator, has been used with chemotherapy regimens in patients who have newly diagnosed osteosarcoma. 

L-MTP-PE, liposomal muramyltripeptidephosphatidyl ethanolamine, was first found to be the most effective agent against microscopic metastases but not against bulky disease in animal studies (Kager et al., 2010). Combining mifamurtide with chemotherapy did not interfere with its immune activity (Kleinerman et al., 1995).

The first adult study of mifamurtide, the INT-0133 study, randomized patients to four different arms at diagnosis as MAP only, MAP in addition to MTP-PE, MAP plus ifosfamide (MAP/I), and MAP plus ifosfamide and MTP-PE. The COG study by Meyers et al., as the continuation of the previous study, found no statistically significant difference in EFS between the four arms although there was a trend towards improved EFS in the arms that contained MTP-PE (p = 0.08) as well as improved OS with statistical significance (78% vs. 70%) (p=0.03) in the mifamurtide group compared to the non-mifamurtide group (Meyers et al., 2008; Kager et al., 2010; Harrison et al., 2017; Roberts et al., 2015; Burgess and Tawbi, 2015). These results led to the approval of this agent in Europe, Mexico, Turkey, and Israel (Harrison and Schwartz, 2017; Roberts et al., 2015; Bishop et al., 2016). In another report, mifamurtide was cost-effective compared with orphan and ultra-orphan drugs (Johal et al., 2013). No long-term side effect of mifamurtide has been reported in the literature (Anderson et al., 2010). The increase in EFS with mifamurtide and the lack of long-term persistent side effects have increased interest on this agent.

The aim of this study was to evaluate whether mifamurtide in addition to chemotherapy improved treatment results in newly diagnosed osteosarcoma cases without metastases. We also wanted to determine its effects on the metastasis and survival rates in the same group. 

Mifamurtide is well-tolerated with minor side effects (headache, pyrexia, chills, tachycardia) (Meyers and Chou, 2014). In our previous study, we reported the side effects of mifamurtide in both metastatic and nonmetastatic osteosarcoma. The most common side effects in all cases were chills and fever. We similarly found the most common side effects in non-metastatic osteosarcoma cases to be fever and chills (Tacyildiz et al., 2018). It was noteworthy that all our patients with headache also had fever. One case also had the side effect of tachycardia that we had not come across in our previous study.

We used the Euramos chemotherapy regimen in addition to mifamurtide as the main treatment. The Euramos-1 study randomized patients with >90% necrosis in the primary tumor that were classified as good responders to receive interferon-2-alpha as maintenance therapy following conventional MAP, and no significant increase was observed in EFS or OS. The same study randomized patients with <90% necrosis in the primary tumor that were classified as poor responders to receive MAP+IE, and again no statistically significant difference was observed for EFS or OS (Harrison et al., 2017; Marina et al., 2016).

We also found similar OS in the MAP and MAP-IE arms (p=0.593), as reported in the Euramos-1 study. The metastasis rate, median time to metastasis and the median survival values were also similar between the two arms.

OS was significantly improved with the addition of mifamurtide to the chemotherapy regimen in non-metastatic osteosarcoma patients in both the INT0331 study and its continuation study of COG. In COG’s study, mifamurtide was found to improve overall survival significantly while other outcome measures were found to be similar to the non-mifamurtide group (Meyers et al., 2008). In our study, the OS rate was 88.9% for all 36 patients at the 43rd month and the addition of mifamurtide did not improve the OS (P=0.652). A longer observation period as in the COG study is required for a more informative statistical evaluation.

In our study, the rates of high-risk factors (bad histologic responder, tumor margin positivity) were similar in our mifamurtide and non-mifamurtide groups. A good histological response for osteosarcoma is defined as <5% viable tumor so tumors with ≤95% necrosis are called bad histologic responders (Provisor et al., 1997). In the group of patients with a necrosis rate ≤95%, the addition of mifamurtide decreased the probability of metastasis development (36.4% vs. 53.9%) regardless of the chemotherapy regimen but this was not statistically significant (p=0.39). A close surgical margin (tumor to surgical margin distance <5 mm) is associated with local recurrence (Chou et al., 2009). Mifamurtide delayed the median time until the development of metastatic disease from 4 months to 7.2 months in the surgical margin positive patients (n=3), considered to be a poor risk group, in our study.

In the COG study, Mayers et al. found no significant difference in median time to disease recurrence with a follow-up period of 6 years (Meyers et al., 2008). We analyzed the patients who experienced metastasis after initial therapy in this study and compared the patients who received and did not receive mifamurtide. For those patients who suffered distant metastasis as a first event, we compared the time from diagnosis in the mifamurtide and non-mifamurtide groups. There was no significant difference in the median time to metastatic disease development (p=0.22) at 43 months. However, the addition of mifamurtide tended to delay the development of distant metastatic disease from a median time of 5 months to 20 months.

Investigation of the other outcome measures in our study showed the primary tumor recurrence, metastasis rate, and survival time to be similar in the mifamurtide and non-mifamurtide groups at 43 months. In case of local recurrence, mifamurtide tended to decrease the rate of metastatic disease.

The limitation of our study is the insufficient number of patients, due to the fact that our cohort included only the non-metastatic patients at diagnosis. The other limitation is the maximum follow-up period of 43 months in the mifamurtide group. Investigation of the other outcome measures showed similar primary tumor recurrence, metastasis rate, and survival time in the mifamurtide and non-mifamurtide groups at 43 months. Mifamurtide tended to decrease the rate of metastatic disease in case of local recurrence.

Chou et al. reported that the addition of MTP for the treatment of patients with metastases at the time of diagnosis did not improve the 5-year EFS (P=0.23) or the 5-year OS (p=0.27) (Chou et al., 2009) from the INT-0133 study. The results in metastatic and recurrent osteosarcoma cases also suggest a decreased risk of recurrence and death with the inclusion of L-MTP-PE in the treatment regimen (Meyers et al., 2008). 

In conclusion, our preliminary data suggest that L-MTP-PE may provide a benefit when added to chemotherapy for the treatment of initially non-metastatic patients with osteosarcoma. However, we need a larger cohort of patients with at least 5 years of follow up. Further studies are required to define the role of MTP-PE in osteosarcoma.

**Table 1:** Euramos Threatment Schedule (16)

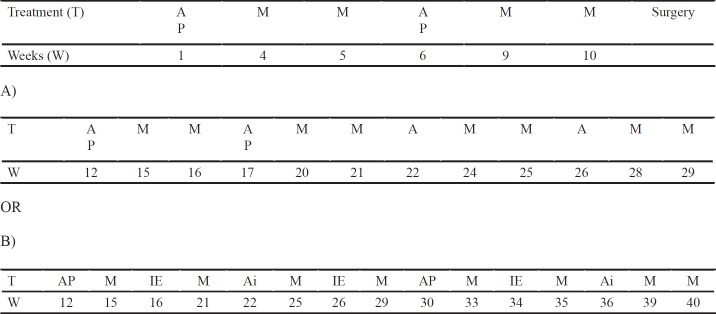

A,Doxorubicin 75 mg/m²/course; P, Cisplatin 120 mg/m²/course; M, Methotrexate 8-12 g/m²/course; E, Etoposide; 500 mg/m²/course; I, Ifosfamide 14 g/m²/cours; İ, Ifosfamide 9 g/m²/course

**Table 2:** Patient Characteristics

	n=36 (%100)	Mifamurtide (+) n=1 (%100)	Mifamurtide (-) n=19 (%100)
Primary tumor site			
Femur	27 (75)	15 (88.2)	12 (63.1)
Tibia	6 (16.7)	2 (11.8)	4 (21)
Other	3 (8.3)	0 (0)	3(15.9 )
Chemotherapy regimen			
Euramos (MAP)	11 (30,5)	6 (35.2)	5 (26.3)
Euramos (MAP+ İE)	14 (38.9)	10 (58.8)	4 (21)
Non-Euramos	11 (30.6)	1 (6)	10 (52.7)
Necrosis			
>95%	9 (27.8)	5 (29.4)	4 (21)
≤95%	24 (66.7)	11 (64.7)	13 (68.4)
Not reported	3 (5.5)	1 (5.9)	2 (10.5)
Surgical Margins			
Negative	30 (83.3)	13 (76.4)	17 (89.5)
Positive	3 (8.3)	2 (11.7)	1 (5,25)
Near the margin (≤1 mm)	1 (2.8)	1 (5.95)	0
Not reported	2 (5.6)	1 (5.95)	1 (5,25)

Abbreviations, MAP, Methotrexate, adriamycin, cisplatin; MAP+ IE, Methotrexate, adriamycin, cisplatin, ifosfamide, etoposide; HDMTX + IE, High dose methotrexate+ ifosfamide+ etoposide

**Table 3:** Necrosis in the Primary Tumor after Induction Chemotherapy

	>95%		≤95%		
	Metastasis + n %	No Metastasis n %	Metastasis + n %	No Metastasis n %	p
Mifamurtide (+)	1 20	4 80	4 36.4	7 63.6	0.39
Mifamurtide (-)	1 25	3 75	7 53.9	6 46.2	

In the group with ≤95 necrosis, mifamurtide has a trend towards decreasing the probability of metastasis (p=0.39). * Huvos grading as modified by the Children’s Cancer Group

## Abbreviations

L-MTP-PE, liposomal muramyltripeptidephosphatidyl ethanolamine.

MAP, high-dose methotrexate, doxorubicin and cisplatin.

IE, ifosfamide and etoposide.

COG, Children’s Oncology Group.

CT, computerized tomography.

PET-CT, positron emission tomography.

HDMTX + IE, High dose methotrexate+ ifosfamide+ etoposide.

Euramos, Doxorubicin 75 mg/m²/course.

A, Methotrexate 8-12 g/m²/course.

M, Cisplatin 120 mg/m²/course.

P, Etoposide 500 mg/m²/course.

E, Ifosfamide 14 g/m²/course.

I, Ifosfamide 9 g/m²/course.
